# Chemoattractant Receptor Homologous to the T Helper 2 Cell (CRTH2) Is Not Expressed in Human Amniocytes and Myocytes

**DOI:** 10.1371/journal.pone.0050734

**Published:** 2012-11-30

**Authors:** Lynne Sykes, Yun Lee, Shirin Khanjani, David A. MacIntyre, Xiao J. Yap, Sathana Ponnampalam, Tiong Ghee Teoh, Phillip R. Bennett

**Affiliations:** 1 Parturition Research Group, Department of Surgery and Cancer, Imperial College London, London, England; 2 Department of Obstetrics and Gynaecology, St Mary’s Hospital, Imperial College Healthcare NHS Trust, London, England; Kaohsiung Chang Gung Memorial Hospital, Taiwan

## Abstract

**Background:**

15-deoxy-Δ 12,14- Prostaglandin J2 (15dPGJ2) inhibits Nuclear factor kappa B (NF-κB) in human myocytes and amniocytes and delays inflammation induced preterm labour in the mouse. 15dPGJ2 is a ligand for the Chemoattractant Receptor Homologous to the T helper 2 cell (CRTH2), a G protein-coupled receptor, present on a subset of T helper 2 (Th2) cells, eosinophils and basophils. It is the second receptor for Prostaglandin D2, whose activation leads to chemotaxis and the production of Th2-type interleukins. The cellular distribution of CRTH2 in non-immune cells has not been extensively researched, and its identification at the protein level has been limited by the lack of specific antibodies. In this study we explored the possibility that CRTH2 plays a role in 15dPGJ2-mediated inhibition of NF-κB and would therefore represent a novel small molecule therapeutic target for the prevention of inflammation induced preterm labour.

**Methods:**

The effect of a small molecule CRTH2 agonist on NF-κB activity in human cultured amniocytes and myocytes was assessed by detection of p65 and phospho-p65 by immunoblot. Endogenous CRTH2 expression in amniocytes, myocytes and peripheral blood mononuclear cells (PBMCs) was examined by PCR, western analysis and flow cytometry, with amniocytes and myocytes transfected with CRTH2 acting as a positive control in flow cytometry studies.

**Results:**

The CRTH2 agonist had no effect on NF-κB activity in amniocytes and myocytes. Although CRTH2 mRNA was detected in amniocytes and myocytes, CRTH2 was not detectable at the protein level, as demonstrated by western analysis and flow cytometry. 15dPGJ2 inhibited phospho-65 in PBMC’S, however the CRTH2 antagonist was not able to attenuate this effect. In conclusion, CRTH2 is not expressed on human amniocytes or myocytes and plays no role in the mechanism of 15dPGJ2-mediated inhibition of NF-κB.

## Introduction

Preterm delivery complicates 6–11% of pregnancies in Europe and North America [1]. Preterm labour is a heterogenous condition [2], however there is a strong association between infection and inflammation, particularly in early preterm deliveries [3]. Despite our increased awareness of this association [4], limited progress has been made in drug development targeted towards anti-inflammatory pathways involved in infection/inflammation induced preterm labour.

Nuclear factor Kappa B (NF-κB) is a transcription factor that plays a central role in controlling the inflammatory response [5]. Human T cell NF-κB activity is suppressed during pregnancy, which likely explains the altered bias from the Th1 to Th2 cytokine ratio [6]. The NF-κB signaling pathway also plays an important role in the process of human labour [7]. Expression of the p65 subunit shows a marked increase in the fundal myometrium during labour [8], associated with an increase in DNA binding activity [9]. NF-κB activity is also increased in amnion during labour [10], with pre-labour samples showing varying degrees of p65 expression, which is likely to reflect the physiological transition that precedes parturition [11]. Many labour associated genes, such as the phospholipases A2 isoenzymes (cPLA2) [12], cyclooxygenase-2 (COX-2), interleukin-8 (IL-8), IL-6, and matrix metalloproteinases (MMPs) [7] are transcriptionally regulated by NF-κB. Pro-inflammatory cytokines such as IL-1β and TNF-α are both regulated by and can activate NF-κB giving rise to a positive feed forward loop and thus further activating the NF-κB regulated genes [13].

Given the importance of NF-κB in the regulation of inflammation and labour, it represents an important potential therapeutic target for the prevention of preterm labour and the neonatal sequale as a direct result of both inflammation and prematurity. We have previously investigated the potential of 15-deoxy Δ^12,14^ Prostaglandin J2 (15dPGJ2), an anti-inflammatory cyclopenentone prostaglandin, in the prevention of inflammation/infection induced preterm labour. 15dPGJ2 inhibits IL-1β stimulated NF-κB in human amniocytes and myocytes, independently of PPAR-γ [14], and reduces the percentage of peripheral blood mononuclear cells producing IFN-γ and TNF-α during pregnancy [15]. Moreover, in a mouse model of lipopolysaccharide-induced preterm labour, 15dPGJ2 delays preterm labour and increases pup survival from 30% to 95% [16]. The precise mechanism of NF-κB inhibition is still not fully understood. 15dPGJ2 is also a ligand for the second Prostaglandin D2 (PGD2) receptor, Chemoattractant receptor-homologous molecule expressed on T helper 2 cells (CRTH2), and binds to it with equivalent affinity to PGD2 as the specific CRTH2 agonist DK-PGD2 [17].

The human CRTH2 gene is located at chromosome 11q12, its transcript length is 2.9 kb consisting of two exons and one intron [18]. It was first named and described by Nagata and colleagues as being expressed on Th2 but not Th1 clones following a gene expression search method [19]. Subsequently it was entitled ‘receptor D2’, a second novel receptor of Prostaglandin D2 (PGD2) [20] as well as ‘GPR44’ following a search for novel G protein coupled receptors [21]. It is now apparent that these are the same receptor, which is also referred to as the cluster differentiation protein CD294 [22]. As a typical GPCR it has seven putative transmembrane domains, with two potential glycosylation sites at its N terminus, and a long cytoplasmic tail with multiple phosphorylation sites [19]. Despite PGD2 acting as a ligand to both the DP1 and CRTH2 receptor, there is a lack of homology between their sequences and their downstream effects differ substantially. CRTH2 is expressed on T helper 2 cells, eosinophils and basophils [23]. The CD4^+^ T cells that express CRTH2 secrete interleukins typical of Th2 cells; IL-4, IL-5, IL-13, and little if any INF-γ [19]. CRTH2 is not expressed on human Th1 cells, and this has led to CRTH2 becoming a cell differentiation marker of Th2 cells [19].

CRTH2 mRNA expression has been described in a number of tissue types, with high expression in heart, brain, stomach, small intestine, liver, thymus, and placenta and moderate expression in the colon, uterus, pancreas, prostate and peripheral blood leukocytes [24]. Other groups have demonstrated CRTH2 mRNA expression at the cellular level in non-immune cells including osteoblasts [25], chondrocytes [26] bronchial epithelial cells [27] and keratinocytes [28]. However, studies of CRTH2 expression at the protein level in non-immune cells are limited, probably due to the lack of specific commercially available antibodies available for detection of CRTH2 using techniques other than flow cytometry. The protein expression profile of CRTH2 in human amniocytes and myocytes has not been investigated to date.

In this study we explored the potential role of CRTH2 in 15dPGJ2 mediated NF-κB inhibition. We examined the effect of a small molecule CRTH2 agonist (Pyl A) on NF-κB activity in human cultured amniocytes and myocytes and examined both the mRNA and protein CRTH2 expression profile in these cell types.

## Materials and Methods

### Ethics Statement

Ethical approval was obtained for placenta (Ref 2002/6283) and myometrium (Ref 3358) from the ethics committees of the Imperial College Healthcare NHS Trust/Imperial College or from the South East London ethical committee for peripheral blood (Ref 10/H0805/54), and in accordance with Imperial College NHS Healthcare Trust Research and Development. Written consent was obtained from all subjects. All clinical investigation was conducted according to the principles expressed in the Declaration of Helsinki.

### Reagents

15dPGJ2 was purchased from Cayman Chemicals (Ann Arbor, MI). The small molecule CRTH2 agonist, Pyl A, was synthesised commercially by Oxygen Healthcare, (Cambridge, London) based on the L-888,607 compound from Merck Frosst Centre for Therapeutic Research (Quebec, Canada) [29]. The CRTH2 antagonist GSKCRTH2X was obtained from Glaxo Smith Kline, (London, UK). IL-1β was from R&D Systems (Abingdon, UK). Antibodies against phospho-p65 (Ser 536), p65 and CRTH2 for western analysis were purchased from Cell Signaling Technology (Beverly, MA), Santa Cruz Biotechnology (Santa Cruz, CA) or Santa Cruz and ProSci Incorporated (Poway, CA) respectively. The β-actin antibody was purchased from Sigma (Gillingham, UK). The flow cytometry antibodies and their isotype controls for CRTH2 were purchased from were from Beckman Coulter (High Wycombe, UK), and CD4 from BD Pharminogen (Oxford, UK). All primers were from Thermo-Scientific (Waltham, MA). For cloning and transfection experiments the sources of reagents were as follows: the intermediate vector pCR® -BluntII-TOPO®, Invitrogen Life Technologies (Grand Island NY); pSG5 expression vector, Agilent Technologies (Wokingham, UK); T7 TNT Coupled Reticulocyte Lysate System, Promega (Madison, WI); GeneJuice® transfection reagent, Novagem (Hertford UK); and AMAXA Basic Nucleofector Smooth Muscle Cell Kit, Lonza (Basel, Switzerland).

### Cell Culture

Placentas and myometrial biopsies were collected at the time of pre-labour caesarean section, and blood was taken from non pregnant women.

#### Preparation of amnion epithelial cells

Amnion was separated from the choriodecidua, cut into strips and washed in phosphate buffered saline (PBS). It was then incubated 0.5 mM of EDTA-PBS for 15 mins at room temperature, and washed in PBS. The intracellular matrix was digested in pre-warmed Dispase at 2 g/L in PBS for 45 mins at 37°C. The epithelial cells were then isolated by shaking the amnion strips vigorously in Dulbecco’s modified Eagle’s medium, DMEM for 3 mins, then pelleted by centrifugation for 10 mins at 2000 rpm. Cells were resuspended in DMEM containing 10% fetal calf serum, 2 mM/L L-glutamine, 100 U/ml Penicillin and 100 µg/ml of Streptomycin and grown to confluence in T25 flasks at 5% CO_2_. Cells were cultured for 24 hours until confluent.

#### Preparation of myocytes

Myometrial biopsies were taken from the upper margin of lower segment incisions during elective caesarean sections. Tissue was washed in PBS and mechanically dissected with two sterile blades to form a paste-like texture. Cells were isolated by incubating with 15 mg of Collagenase 1A, 15 mg of Collagenase X and 50 mg of BSA in 30 mls of PBS for 45 mins at 37°C. The suspension was filtered through a cell strainer and centrifuged at 400 *g* for 5 mins. The cells were resuspended and cultured in DMEM as described above. Myocytes were cultured until confluent. Passage one myocytes were used for transfection studies and passage 0–4 for studies of endogenous CRTH2.

#### Preparation of placenta and choriodecidua

A biopsy of placenta and choriodecidua were snap frozen and ground in liquid nitrogen in preparation for mRNA extraction.

#### Preparation of peripheral blood mononuclear cells

Blood was diluted 1∶1 with phosphate buffered saline (PBS) and carefully layered onto Ficoll-Paque™ PLUS (GE Healthcare, Uppsala, Sweden) before centrifuging at 400 *g* for 40 mins at room temperature. After centrifugation, the halo containing PBMCs was carefully transferred into a clean centrifuge tube and washed twice with 7 ml of PBS. After centrifugation (400 *g* for 10 mins), the cell pellet was resuspended in extraction buffer or RPMI 1640 culture medium (Invitrogen Life Technologies, Grand Island, NY) culture medium.

### Cell Treatment

A dose response with the CRTH2 agonists 15dPGJ2 and Pyl A from 0.1 µM-32 µM for 2 hrs was used to determine the effect on NF-κB activity in IL-1β treated amniocytes and myocytes. Stimulation was required with 1 ng/ml of IL-1β, since basal activity in pre-labour amnion and myometrium is low [14]. Based on previous work with 15dPGJ2 on amniocytes, myocytes [14] and PBMC’s [15], 32 µM of 15dPGJ2 was used for determining the effect of NF-κB activity in PBMC’s. Prior to treatment with 15dPGJ2, cells were pre-treated with 2 µM of GSKCRTH2X (a CRTH2 antagonist) or vehicle for 45 mins.

### PCR

Total RNA was extracted using TRIzol® (Invitrogen) and reverse transcribed by Superscript III (Invitrogen). Taq Po was used for qualitative PCR. CRTH2 was detected using qualitative PCR with the Primer sets A and B (Table 1) under cycling conditions of; 95°C for 3 min, amplification by 35 cycles of PCR (94°C, 1 min; 67°C, 1 min; 72°C, 2 min) for set A and 95°C for 3 min, amplification by 40 cycles of PCR (94°C, 1 min; 63°C, 1 min; 72°C, 2 min) for set B with AB1 StepOne. Products of quantitative PCR were also qualitatively assessed on an agarose gel having been amplified with primer set C (Table 1) with SYBRGreen PCR Master mix (Applied Biosystems) under the following cycling conditions of activation at 50°C for 2 min with 40 cycles of (95°C, 1 min; 95°C, 15 s; 60°C, 1 min). Peripheral mononuclear blood cell cDNA was used to amplify a 1.188 kb CRTH2 transcript using primer set D with activation at 95°C for 3 min and 36 cycles of (94°C, 1 min; 75°C, 1 min; 72°C, 2 min). The primer sets produced amplicons where the intron/exon boundary was crossed wherever possible. Non-template controls and reverse transcriptase negative controls were used. Products were subjected to gel electrophoresis and detected by staining with Sybersafe (Invitrogen) to assess for correct product size.

**Table 1:** The primer sequences used for amplification of CRTH2.

	Forward 5′	Anti-sense 5′	bp
Set A	CCTCTGTGCCCAGAGCCCCACGATGTCGGC	CACGGCCAAGAAGTAGGTGAAGAAG	309
Set B	CTGCCCTTCTTCACCTACTTCTTGGC	GTGTCCCGGAACACGAAATAGGGCAC	265
Set C	CCTCTGTGCCCAGAGCCCCACGATGTCGGC	ATGTAGCGGATGCTGGTGTTG	114
Set D*	5′GGATCCATGTCGGCCAACGCCACACTGAAGCC (*Includes BAMH I site)	AGATCTCTAACTCGAGGTGCTGCTCAGCGCCC (*Includes BGL II site)	1188

### Sodium Dodecyl Sulfate Polyacrylamide Gel Electrophoresis (SDS-PAGE) and Western Blotting

Protein was extracted from cells with whole cell lysis buffer (Cell Signalling, Beverly, MA) and 5 µl/ml of protease inhibitor (Sigma). Cells were incubated with ice cold buffer for 5 mins and centrifuged for 20 mins at 13,000 rpm at 4°C. Protein extraction from tissue was performed by homogenising in whole cell lysis buffer prior to the above incubation steps. For nuclear protein extraction cells were lysed in Buffer A (10 mM HEPES, 10 mM KCL, 0.1 mM EGTA, 0.1 mM EDTA, 1 mM DTT, 1%(v) NP-40 with protease inhibitor), with the cytosolic fraction collected following centrifugation at 13,000 rpm for 60s at 4°C. The pellet was then resuspended in Buffer B (10 mM HEPES, 10 mM KCL, 0.1 mM EGTA, 0.1 mM EDTA, 2MM DTT, 400 mM NaCl and 1% v/v NP-40 and protease inhibitors), incubated on a shaker for 15 mins at 4°C, and then centrifuged at 13,000 rpm for 5 mins at 4°C. Prior to SDS-PAGE, protein concentrations were determined using the BIORAD quantification assay measuring absorbance at 655 nm. Between 15 and 50 µg of extracted protein per sample, was resolved by SDS-PAGE and subsequently transferred onto polyvinyl difluoride (PVDF) membranes (GE Healthcare) at 100 V constant at 4°C. Following transfer, the membrane was then blocked in 5% (wt/vol) milk in tris-buffered saline (TBS) supplemented with 0.1% Tween 20 (TBST) for 1 h. The membrane was then probed with the relevant primary antibody followed by the appropriate secondary antibody under conditions specified in (Table 2). Chemiluminescence detection was then carried out with ECL Plus (GE Healthcare) and the membranes developed using a high Performance chemiluminescence film (GE Healthcare). Blots were scanned and densitometry was performed with ImageJ (v1.44p).

**Table 2:** The incubation conditions and the catalogue numbers for all antibodies used.

	Primary antibody	Secondary antibody	Cat number
P-p65 (Ser536)	1∶1000 Overnight at 4°C	Anti-rabbit; 1∶2000 1 hr RT	Cat 3033s
p65	1∶1000 Overnight at 4°C	Anti-mouse; 1∶200 1 hr RT	Cat no 8008
CRTH2	1∶200 Overnight at 4°C	Anti-goat: 1∶2000 1 hr RT	Cat no. 23092
CRTH2	1∶500 Overnight at 4°C	Anti-rat; 1∶3000 1 hr RT	Cat no. 2178
CRTH2	1∶500 Overnight at 4°C	Anti-rabbit; 1∶2000 1 hr RT	Cat no. PR-4029
β-actin	160∶000 30 minutes at RT	Anti-mouse; 20∶1000 15 mins RT	Cat no. A 2228

### Cloning

Human peripheral leukocytes were used to extract mRNA and to reverse transcribe to cDNA with random hexamers with Superscript III according to manufacturer’s instructions (Invitrogen Life Technologies). A 1.18 kB CRTH2 transcript of the coding region was amplified with primers which introduced BamH I digest sequence upstream of the CRTH2 coding sequence and a BGL II restriction digest sequence downstream (Table 2). The transcript was cloned into an intermediate TOPO Vector (pCR® -BluntII-TOPO®) according to manufacturer’s instructions (Invitrogen Life Technologies). The plasmid was digested at the BamH1 and BglII sites, run on a 1% DNA Agarose gel, cut and purified using the Geneclean kit for insertion into the pSG5 expression vector. Plasmids were digested and the insert was purified for sequencing to confirm that CRTH2 was present and in frame. The pSG5 expression vector was selected since this has previously been shown to lead to high and stable expression of multiple proteins in myocytes [30].

### In vitro Transcription Translation and Transfection

CRTH2 expression in a cell free system was examined with the T7 TNT Coupled Reticulocyte Lysate System. The TNT kit was used as per manufacturer’s instructions (Promega) to produce CRTH2 with both cold Methionine and radioactive ^35^S Methionine for detection by both western analysis and x-ray film respectively. The progesterone pSG5 expression vector, a kind donation of Dr Yun Lee, served as a positive control for the efficiency of the transcription-translation reaction. For transfection of CRTH2 into amniocytes; primary amnion epithelial cells were cultured until 80% confluence. GeneJuice® transfection reagent was used according to manufacturer’s guidance (Novagem) with 1 µg of DNA per well. For transfection of CRTH2 into myocytes; myometrial smooth muscle cells were cultured until 100% confluence. The AMAXA Basic Nucleofector Smooth Muscle Cell Kit was used as per manufacturer’s instructions (Lonza). Electroporation was performed with the Nucleofector Program using Program A-033. GFP was used as a positive control for the transfection efficiency. Gene expression of Green Fluorescent Protein (GFP) was determined by fluorescent microscope on days 1 and 2 post transfection. Gene expression of CRTH2 was determined by Flow cytometry on days 1 and 2 post transfection.

### Flow Cytometry for Detection of CRTH2

PBMC, amniocyte and myocyte pellets were resuspended in staining buffer (1% Fetal Calf Serum, 0.09% Sodium Azide in PBS). Cells were incubated in the dark for 1 h at 37°C with 20–40 µl of CRTH2-PE. PBMCs were also incubated with 3 ul of CD4-APC. Mouse IgG1 κ –APC and Rat IgG_2a_-PE were used as isotype controls. After incubation, the PBMC suspension was washed twice in 1 ml of PBS and then resuspended in PBS for analysis. The FACSCalibr flow cytometer was used for CRTH2 detection and settings were as follows: PBMCs; Forward scatter E0 Voltage, 1.00 Amp gain Lin, and Side scatter of 329 Voltage, 1.00 Amp gain Lin, amniocytes; Forward Scatter E1; Voltage 4.77; Amp gain Lin; Side scatter of 320; Voltage 1.00; Amp gain Lin; FL2 347 log, Myocytes; Forward Scatter E1; Voltage 4.37; Amp gain Lin; Side scatter of 197; Voltage log; FL2 350 log. Results are expressed as percentage of cells expressing the receptor and mean fluorescence intensity of receptor expression. The geographical mean was used since data was collected on a logarithmic scale and is a better indicator of the central tendency of the population.

### Statistical Analysis

Experimental sample groups consisted of 3–6 biological replicates. Statistical analysis was performed with Graph-Pad Prism (v5.0; GraphPad Software, San Diego, CA). ANOVA of repeated measures was conducted where appropriate, with Bonferroni’s multiple comparison’s test for *post-hoc* analysis. Samples with *P*<0.05 was considered to be statistically significant.

## Results

### The CRTH2 Agonist 15dPGJ2 Inhibits IL-1β Induced NF-κB Activity in Human Amniocytes and Myocytes but the CRTH2 Agonist Pyl A does not Replicate this Effect

**Figure 1:**
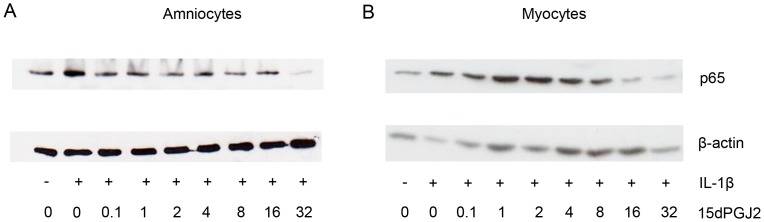
15dPGJ2 reduced NF-κB p65 activity in amniocytes and myocytes. Protein was extracted from IL-1β stimulated and 15dPGJ2 treated cells and levels of nuclear p65 were examined using immunoblotting. A dose response of 0.1–32 µM of 15dPGJ2 was used (n = 3). Representative immunoblots are shown for amniocytes, (**A**), myocytes (**B**). Immunoblots were re-probed for β-actin as an internal loading control.

**Figure 2:**
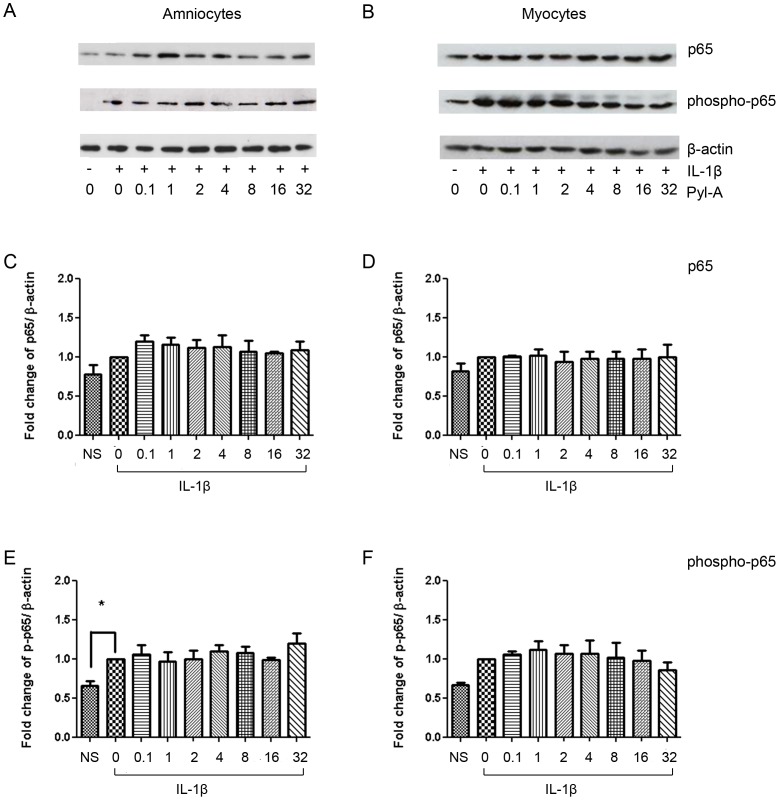
Pyl A has no effect on NF-κB p65 activity in amniocytes and myocytes. Protein was extracted from IL-1β stimulated and Pyl treated cells and levels of nuclear p65 and phosphorylated p65 (p-p65) were examined using immunoblotting. A dose response of 0.1–32 µM of Pyl A was used. Representative immunoblots are shown for amniocytes, (**A**) and myocytes (**B**). Immunoblots were re-probed for β-actin as an internal loading control. Densitometric analysis of the immunoblots was conducted revealing no effect of Pyl A on p65 or p-p65 in amniocytes (**C**, **E**) or myocytes (**D**, **F**). NS = non-stimulated (non-IL-1β treated cells). Effect of treatment was examined for statistical significance using ANOVA of repeated measures with Bonferroni’s multiple comparison test; *P<0.05.

We have previously shown that 15dPGJ2 inhibits IL-1β induced NF-κB activity in human amniocytes and myocytes in a mechanism independent of PPAR-γ [14]. Here we initially replicated these results showing a significant reduction in IL-1β induced NF-κB, as determined by nuclear p65, to below basal levels in amniocytes at 32 µM and in myocytes from 16 µM (Figure 1A and 1B). If CRTH2 is expressed in amniocytes and myocytes and is the mechanism of action of 15dPGJ2, we would expect a small molecule CRTH2 agonist to replicate the effect of 15dPGJ2 We therefore examined the effect of the CRTH2 agonist Pyl A on IL-1β induced NF-κB in amniocytes and myocytes to determine if the small molecule agonist could replicate the effects of 15dPGJ2. NF-κB activity was assessed by examining nuclear p65 and phospho-p65 by immunoblot. There was no difference in IL-1β stimulated NF-κB activity seen with Pyl A treatment in either cell type. Representative immunoblots for amniocytes and myocytes are shown in Figures 2A and 2B respectively, with densitometric analysis taking into account β-actin loading controls seen in Figures 2C–F.

### CRTH2 mRNA is Expressed in Amniocytes and Myocytes

**Figure 3:**
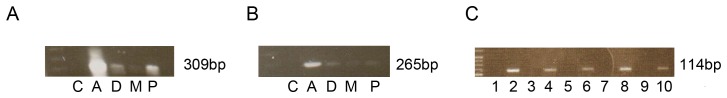
CRTH2 mRNA is expressed in amniocytes and myocytes. mRNA was isolated from cultured amniocytes, myocytes, PBMCs, choriodecidual and placental extracts and converted to cDNA (n = 6). Qualitative PCR was used with three primer sets to amplify CRTH2 showing product sizes of 309 bp, 265 bp, and 114 bp. Non-template and reverse transcriptase negative controls were used and mRNA from placenta, choriodecidua and peripheral blood mononuclear cells were used as a positive controls. (**A**,**B**): Non-template control (lane 1), amniocytes (lane 2), choriodecidua (lane 3), myocytes (lane 4), placenta (lane 5); (**C**): Reverse transcriptase controls (lanes 1,3,5,7,9), PBMCs (lane 2), amniocytes (lanes 4,6), myocytes (lanes 8,10).

The absence of Pyl A mediated inhibition of NF-κB in amniocytes and myocytes led us to question if this was due to a lack of expression of CRTH2 in these cells, or, alternatively that there is no role for CRTH2 in 15dPGJ2 mediated inhibition of NF-κB. Accordingly, CRTH2 mRNA and protein expression of in amniocytes and myocytes was examined by PCR and western analysis. CRTH2 mRNA was detected in amniocytes and myocytes as well as choriodecidual tissue and placental tissue with two primer sets. Placenta served as a positive control [24] and non-template negative controls were included (Figure 3A and 3B). A third primer set was used as confirmation of consistent expression in amniocytes and myocytes with reverse transcriptase negative controls and PBMCs as a positive control (Figure 3C). N = 6 biological replicates were examined.

### CRTH2 is not Expressed at the Protein Level in Amniocytes and Myocytes

#### Western analysis of endogenous CRTH2

**Figure 4:**
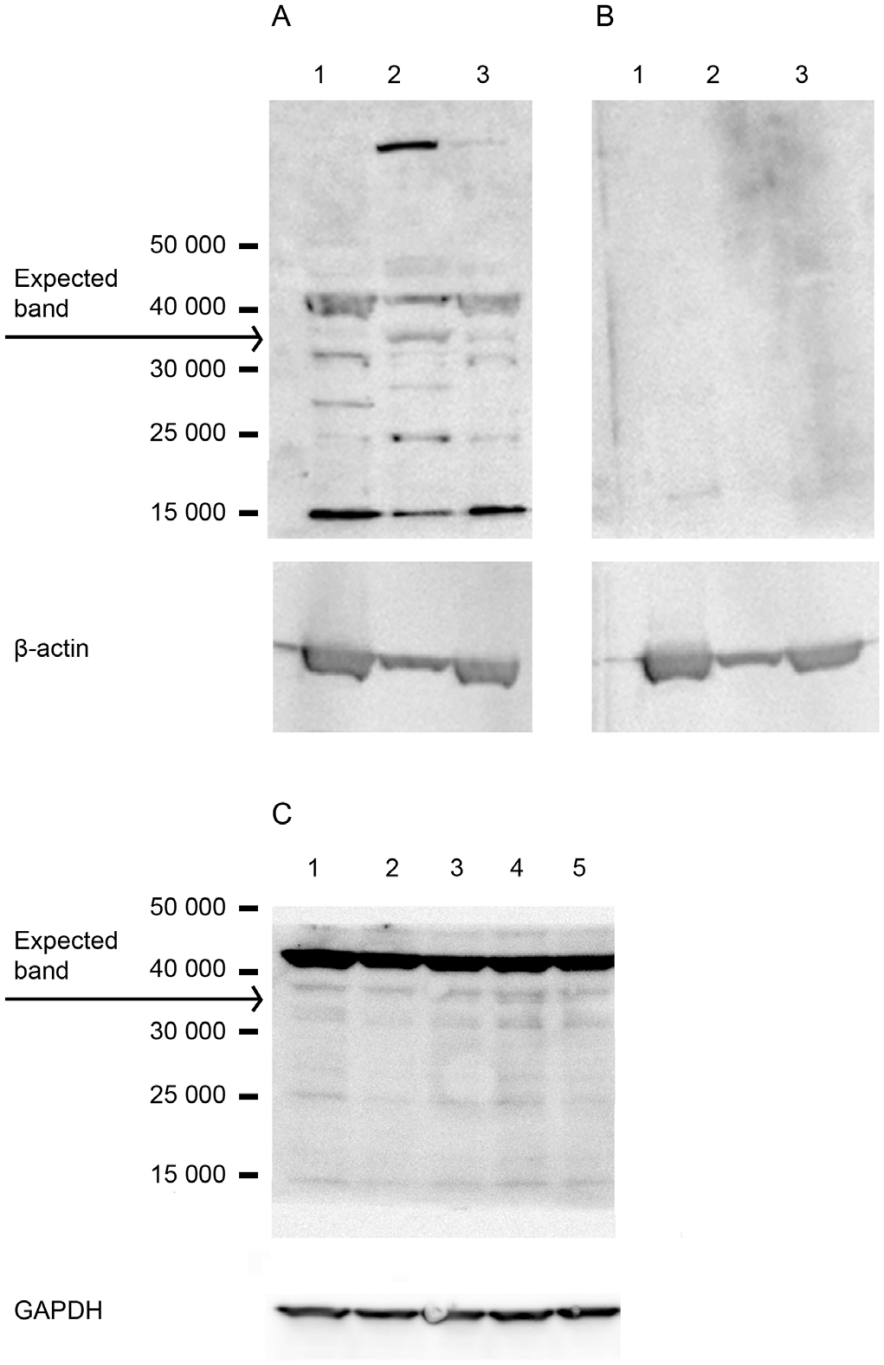
Western blot of protein lysate from PBMCs, amniocytes, myocytes. 50 µg of whole cell lysate from PBMCs, amniocytes, myocytes were subject to western analysis. Membranes were probed with the primary antibody -SC-23092 to detect a Mr∼34 000 product (**A**; Lanes 1–3). The secondary antibody control blot is also shown (**B**; Lanes 1–3). β-actin was used as a loading control. Lane 1 = PBMCs, lane 2 = amniocytes, lane 3 = myocytes. Multiple bands are seen on the blot. A band appears at Mr∼34 000 in amniocytes, faintly in the myocyte lane, but is absent in the positive control PBMCs lane. However, the strongest bands appear at Mr∼15 000 and at just above Mr∼43 000 in all lanes. Myocytes from passage zero, one, two, three and four (lanes 1–5 respectively) were also examined for CRTH2 expression showing a very faint band at Mr∼34 000, with no effect of passage number (**C**).

CRTH2 expression at the protein level was then examined by immunoblot using the CRTH2 antibody Sc-23092 (Figure 4). 50 µg of total protein derived from PBMC whole cell lysate, amniocytes, myocytes was used. Multiple bands are seen in both the positive control (PBMCs) and in the amniocyte and myocyte lanes. A band appears at Mr∼34 000 in amniocytes, faintly in the myocyte lane, but is absent in the positive control PBMCs lane. However, the strongest bands appear at M_r_∼15 000 and at just above M_r_∼43 000 in all lanes. No culture dependent effect was seen as demonstrated by myocytes from passage 0–4 in panel C, Figure 4. With the absence of CRTH2 detection in the positive control, we cannot determine the presence or absence of endogenous CRTH2 in amniocytes and myocytes from this immunoblot alone.

#### Detection of CRTH2 in the pSG5 expression vector

**Figure 5:**
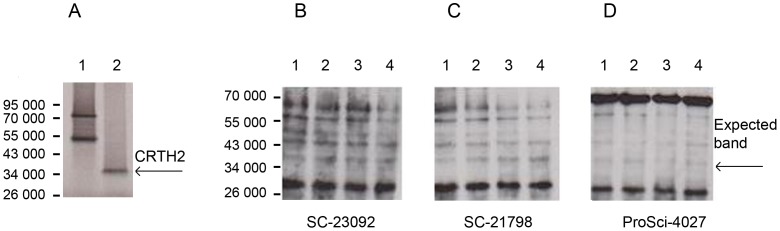
Detection of radiolabelled CRTH2 in pSG5 expression vector by x-ray and immunoblot. The in vitro transcription translation kit was used with 35^S^ Methionine to demonstrate the expression of CRTH2 and to provide a positive control for detection of CRTH2. A Mr∼34 000 product was detected by x-ray (Lane 2). The progesterone expression vector was used as a control for the TNT kit (**A**). The protein lysate from the TNT kit was subjected to western analysis; Negative Control: TNT kit with no plasmid DNA (lane 1), Negative control: Progesterone PSG5 Expression vector (lane 2), Positive control: 35^S^ Methionine labelled CRTH2 (Lane 3), Cold Methionine CRTH2 protein product (lane 4). Membranes were probed with 3 commercial antibodies; SC-23092 (**B**), SC-21798 (**C**), ProSci 4027 (**D**). None of the antibodies detected CRTH2.

With the possibility of very low endogenous protein expression of CRTH2 explaining the reason for the antibody failing to detect CRTH2 in the positive control of the immunoblot in Figure 4, we cloned and synthesised CRTH2 using an *in-vitro* transcription translation kit to form a highly concentrated CRTH2 protein lysate to serve as a positive control. A band at M_r_∼34 000, consistent with the reported expected size could be clearly observed following detection of radioactive ^35^S Methionine by x-ray film (Figure 5A). The protein lysate was then loaded and transferred onto immunoblots and probed with three commercially available antibodies designed for western blotting. No band was detected at M_r_∼34 000, with non-specific bands appearing also in the negative control of the TNT kit without plasmid DNA (Figure 5B–D).

#### Detection of endogenous CRTH2 by flow cytometry

**Figure 6:**
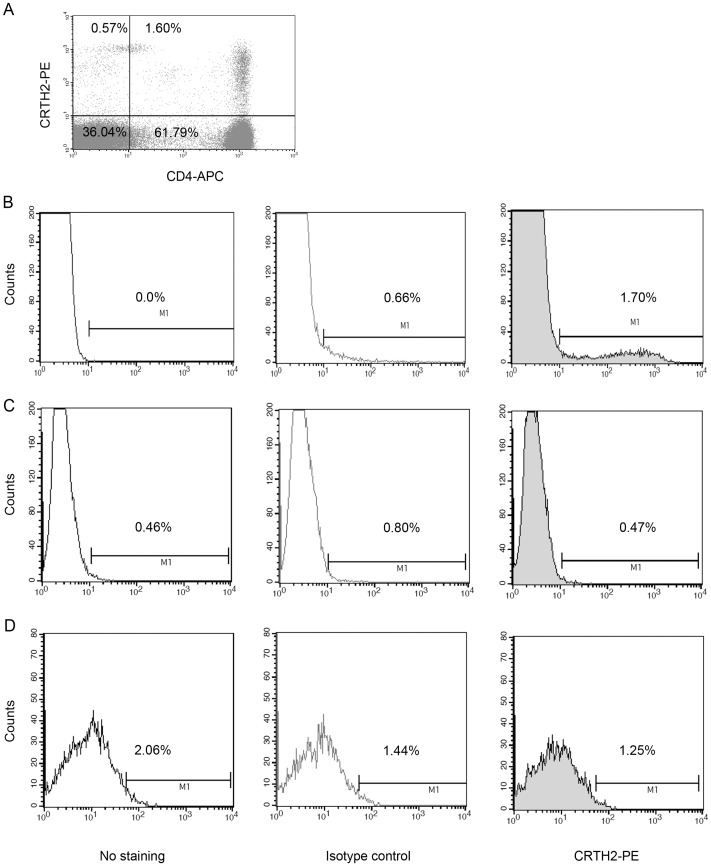
Flow cytometry for the detection of endogenous CRTH2. Lymphocytes were gated according to forward scatter and side scatter and T helper cells were identified using CD4 as a cell surface marker (n = 6). A representative cytogram is presented with the right upper quadrant showing CRTH2^+^/CD4^+^ lymphocytes (**A**). Histograms showing no staining, isotype control and CRTH2^+^ labelled lymphocytes (**B**), amniocytes (**C**) and myocytes (**D**), (n = 5).

Several studies have used commercially available antibodies for flow cytometry successfully for the detection of endogenous CRTH2 [27], [31], [32]. Similarly, we were able to detect endogenous CRTH2 by flow cytometry on human lymphocytes. Lymphocytes were gated based on forward and side scatter profile. A representative cytogram is shown with CRTH2^+^/CD4^+^ cells seen in the right upper quadrant (Figure 6A). 1.6% of lymphocytes were CRTH2^+^/CD4^+^, and 0.57% were CRTH2^+^/CD4^−^. A representative histogram of cells with no staining, isotype control and CRTH2 staining is shown, with the mean fluorescence intensity of 157.80 compared to 22.8 in the isotype control sample (Figure 6B). No CRTH2 expression was seen in amniocytes or myocytes, with a mean fluorescence intensity of 14.41 with labelling compared to 15.71 for the isotype in amniocytes; and 69.69 with labelling compared to 69.30 in the isotype control labelled myocytes (Figure 6C and 6D), n = 6. Table 3 summarises the mean fluorescence intensity and percentage of cells expressing CRTH2 in the gated populations.

**Table 3:** An overview of the mean fluorescence intensities and percentages in the gated cells during detection of endogenous CRTH2 in lymphocytes, amniocytes and myocytes.

Endogenous expression	No stain	Isotype-PE	CRTH2-PE
Lymphocytes	12.98 (MFI) 0.0 (%)	22.8 (MFI) 0.66 (%)	157.80 (MFI) 1.70 (%)
Amniocytes	13.91 (MFI) 0.46 (%)	15.71 (MFI) 0.80 (%)	14.41 (MFI) 0.47 (%)
Myocytes	71.38 (MFI) 2.06 (%)	69.30 (MFI) 1.44 (%)	69.69 (MFI) 1.25 (%)

#### Detection of transfected CRTH2 in amniocytes and myocytes by flow cytometry

**Figure 7:**
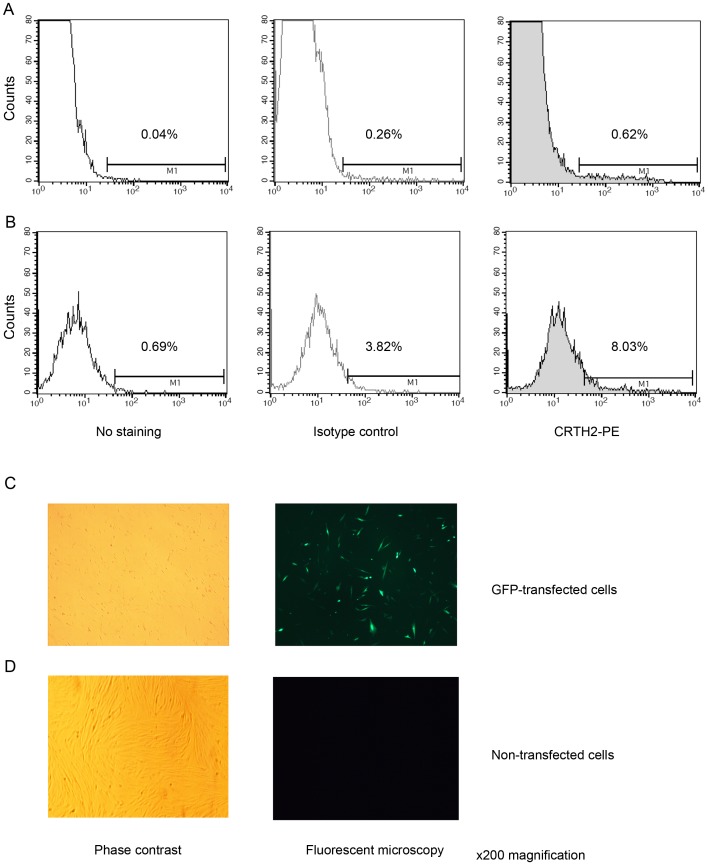
Flow cytometry for the detection of transfected CRTH2. Amniocytes (**A**) and myocytes (**B**) were transfected using gene juice and electroporation respectively with CRTH2 PSG5 expression vector and labeled with anti-CRTH2-PE. A small shift to the right is seen in the histogram showing expression of CRTH2 in transfected cells with an increase in the mean fluorescence intensity from 87.75 in the isotype control to 143.49 in amniocytes, and from 68.84 to 96.97 in myocytes. An example of a GFP control is given for the myocyte transfection, with cells imaged with phase contrast and with the fluorescent microscope to detect GFP transfected cells (**C**). A direct comparison is also shown with non-GFP transfected cells (**D**).

Since we could not detect CRTH2 in amniocytes or amniocytes by flow cytometry we transfected in a CRTH2 expression vector to confirm that endogenous CRTH2 could have been detected if present. CRTH2 was detected in amniocytes and myocytes transfected with CRTH2, as seen by an increase in the mean fluorescence intensity. Representative histograms are shown for transfected samples, with isotype controls for amniocytes (Figure 7A) and myocytes (Figure 7B). The mean fluorescence intensity increased from 87.75 in the isotype control to 143.49 in amniocytes, and from 68.84 to 96.97 in myocytes. A GFP transfection control is shown to confirm adequate transfection efficiency (Figure 7C). Table 4 summarises the mean fluorescence intensity and percentage of cells expressing CRTH2 in the gated populations.

**Table 4:** An overview of the mean fluorescence intensities and percentages in the gated cells during detection of transfected CRTH2 in amniocytes and myocytes.

Transfected expression	No stain	Isotype-PE	CRTH2-PE
Amniocytes	45.20 (MFI) 0.04 (%)	87.75 (MFI) 0.26 (%)	143.49 (MFI) 0.62 (%)
Myocytes	63.88 (MFI) 0.69 (%)	68.84 (MFI) 3.82 (%)	96.97 (MFI) 8.03(%)

### 15dPGJ2 Inhibits Basal NF-κB Activity in PBMC’s in a Mechanism Independent of CRTH2

**Figure 8:**
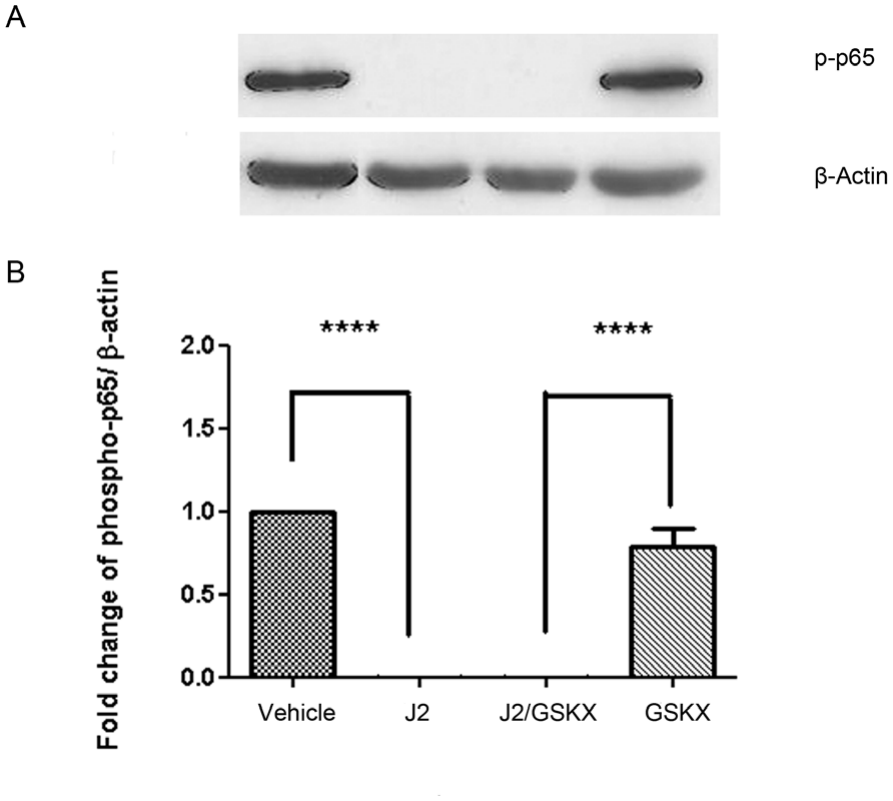
The effect of 15dPGJ2 on basal NF-κB p65 phosphorylation in peripheral blood mononuclear cells. PBMCs were treated with 32 µM of 15dPGJ2 for 2 hours with or without preincubation with 2 µM of GSKCRTH2X for 45 mins. Whole cell protein lysate was examined for levels of phosphorylated p65 (p-p65) using immunoblotting. A representative immunoblot from n = 6 samples are shown (**A**). Immunoblots were reprobed for β-actin as an internal loading control. Densitometric analysis of the immunoblots was conducted revealing a complete inhibition of p-p65 levels with 15dPGJ2. Pre-incubation with the CRTH2 antagonist GSKCRTH2X had no effect on inhibition (**B**). J2 = 15dPGJ2, GSKX = GSKCRTH2X. Effect of treatment was examined for statistical significance using ANOVA of repeated measures with Bonferroni’s multiple comparison test; **** *P*<0.0001.

Thus far we have demonstrated that CRTH2 is not expressed at detectable levels in amniocytes and myocytes. We therefore proposed that CRTH2 does not play a role in 15dPGJ2-mediated inhibition of NF-κB. To confirm this we treated PBMCs with 15dPGJ2 with or without the CRTH2 antagonist, GSKCRTH2X (n = 6). 15dPGJ2 showed complete inhibition of phospho-65 (*P<0.0001*) which was not attenuated by pre-incubation with the CRTH2 antagonist (Figure 8).

## Discussion

Preterm birth accounts for 70–75% of neonatal morbidity and mortality [33]. In 80–85% of cases of spontaneous preterm labour <28 weeks there is evidence of intrauterine infection [34]. The presence of inflammation can increase the risk of neonatal morbidity, independently of prematurity [35]. Inflammatory cytokines, produced in association with infection, can activate the transcription factor NF-κB. Since NF-κB controls the expression of many labour associated genes and pro-inflammatory cytokines, targeting the inhibition of NF-κB seems attractive to prevent both infection induced preterm labour and the associated risk of brain injury associated with inflammation. In this study we explored the possibility that CRTH2 plays a role in 15dPGJ2-mediated inhibition of NF-κB both at a local level in cells of the maternal fetal interface; amniocytes and myocytes, and systemically in peripheral blood mononuclear cells. If this were true, then small molecule agonists of CRTH2 could potentially be used in the management of preterm labour.

We have previously shown that 15dPGJ2 inhibits IL-1β induced NF-κB activity in human amniocytes and myocytes through a mechanism independent of the PPAR-γ receptor [14]. 15dPGJ2 is a CRTH2 agonist, and upregulates CD11b expression in eosinophils via CRTH2 [36]. This led us to examine the effect of a small molecule CRTH2 agonist, Pyl A, on NF-κB activity in human amniocytes and myocytes. We saw no effect of Pyl A on IL-1β stimulated p65 or phospho-p65 in either cell type (Figure 2). We have also previously seen differing effects between 15dPGJ2 and Pyl A on NF-κB activity in the murine myometrium. Intrauterine administration of 15dPGJ2 inhibits LPS induced NF-κB in the murine myometrium and is associated with a delay in preterm labour [16]. In contrast, Pyl A did not inhibit NF-κB but instead augmented LPS stimulated NF-κB, which led to earlier preterm delivery of the pups (unpublished data). Despite both 15dPGJ2 and Pyl A activating the CRTH2 receptor, cross talk may exist between their other downstream targets of varying cell types, which could explain their contrasting effects when given *in vivo*.

Few studies have sought to characterise CRTH2 expression in human gestational tissues. Helliwell et al examined mRNA expression in amnion, choriodecidua and placental tissue [37]. They failed to detect CRTH2 in all 24 amnion samples, both preterm and term. 3/12 preterm choriodecidual samples expressed CRTH2, all three had histological evidence of choriamnionitis, whereas 7/12 term samples expressed CRTH2. All placental samples had detectable but variable levels of CRTH2 mRNA, with no difference between preterm and term samples. Nagata et al have reported detectable CRTH2 mRNA in many human tissue types including the placenta and non-pregnant myometrium [24]. Since CRTH2 is classically expressed on immune cells, it is plausible that the mRNA detected in these tissue samples is from infiltrating leukocytes, which would explain why CRTH2 can be detected choriodecidual samples associated with chorioamnionitis.

Several studies have examined the presence of CRTH2 positive T cells in the decidua in early pregnancy in association with the potential CRTH2 mediated bias towards the anti-inflammatory Th2 cytokine profile at the maternal fetal interface [38], [39], [40]. The percentage of CRTH2^+^ cells in CD4^+^/CD3^+^ cells are higher in the decidua than the peripheral blood in early pregnancy [38]. It has been proposed that decidual Th2 cells are recruited to the maternal fetal interface by PGD2 mediated chemoattraction via the CRTH2 receptor [39]. In this study we examined CRTH2 at the cellular level and found detectable levels of CRTH2 mRNA in amniocytes and myocytes with three primer sets (Figure 3), using choriodecidua and placental tissue and PBMCs as positive controls. Expression levels were low with cycle numbers of between 35 and 40 used for detection. This is consistent with the low expression levels seen in other cell types such as monocytes and dendritic cells, being amplified with up to 40 cycles [41].

To determine if the mRNA was translated into protein in amniocytes and myocytes we used a polyclonal antibody against the extracellular domain of CRTH2 intended for detection of human CRTH2 at Mr∼34 000 by immunoblot. This antibody failed to convincingly detect CRTH2 in amniocyte and myocyte protein lysate (Figure 4). Although a faint band was detected in amniocytes and myocytes, no band was detected in the positive control (PBMCs). Since no band was detected in the positive control, we cannot conclude with this immunoblot if CRTH2 is endogenously expressed or not. There are relatively few studies that have used western analysis for the detection of human CRTH2 by western analysis. Zayed et al detected bands at Mr∼41 000 and Mr∼55 000 for chondrocytes, using osteoblasts as a positive control [26]. However, they did not include blocking or secondary antibody controls to confirm specificity. CRTH2 has previously been demonstrated in osteoclasts by immunohistochemistry with appropriate secondary antibody controls [42]. However, sale of this antibody has since been discontinued because of non-specificity. Colombe et al detected a band for CRTH2 in keratinocytes and melanocytes at Mr∼45 000 with the same antibody, again without negative or secondary antibody controls [28].

We cloned CRTH2 to provide a highly concentrated positive control for detection by western analysis and flow cytometry. CRTH2 was synthesized at the correct size of Mr∼34 000 (Figure 5A), conforming roughly with the Kozak rule, and consistent with the size determined by Nagata et al [19]. Nagata et al raised three antibodies against CRTH2 which detected the presumed glycosylated form of CRTH2 at Mr∼55 000 and the Mr∼34 000 CRTH2 product following oligosaccharide cleavage with Endo F, consistent with CRTH2 carrying N-glycosylation sites as predicted by its amnio acid sequence.

Three commercially available antibodies failed to detect in vitro translated CRTH2 by immunoblot (Figure 5B–D). CRTH2 has 7 transmembrane domains which is typical of the G protein coupled seven transmembrane receptor superfamily. There are two potential sites for N-glycosylation in the first extracellular domain, and four consensus sites for protein kinase c phosphorylation on a long cytoplasmic tail [19]. It is very likely that the antibodies require the correct folding structure and/or post translational modifications of the CRTH2 protein to bind to their epitopes. The limitation of the cell free system is the inability to construct the protein to its correctly folded structure or to reproduce the post-translational modifications that exist in the cell, which may explain why CRTH2 was not detected.

To overcome the limitation of the cell free system, we transfected CRTH2 into amniocytes and myocytes. There are a number of studies in which flow cytometry successfully detected human CRTH2 on immune cells [19], [31], [32], [38], [43], [44]. Since no conclusion could be made on CRTH2 expression in amniocytes and myocytes by immunoblot, we used flow cytometry to examine CRTH2 expression on amniocytes and myocytes, using PBMCs as a positive control. We used a monoclonal antibody raised against the BM16 clone which has also successfully detected CRTH2 on non immune cells; human bronchial epithelial cells [27]. The expression of CRTH2 is low in known cellular populations, with between 0.4–6.5% of peripheral blood CD4^+^ cells expressing the receptor [19]. Our data is consistent with this, with the representative sample showing 2.5% of CD4^+^ cells expressing CRTH2, (Figure 6B). This antibody did not detect endogenous CRTH2 in amniocytes and myocytes (Figure 6C and 6D). However, CRTH2 was detected at low levels in transfected cells (Figure 7A and 7B). The pSG5 expression vector leads to high expression of multiple proteins in myocytes [30], and good transfection efficiency was achieved as demonstrated by the GFP control (Figure 7C). Therefore, the inefficiency of amniocytes and myocytes to express a stable CRTH2 protein supports our conclusion that CRTH2 is not endogenously expressed in amniocytes and myocytes.

The absence of CRTH2 on human amniocytes and myocytes imply that the mechanism of 15dPGJ2 mediated inhibition of NF-κB is independent of CRTH2. Moreover, the CRTH2 antagonist failed to attenuate the effects of 15dPGJ2 in peripheral blood mononuclear cells confirming that CRTH2 plays no role in NF-κB inhibition (Figure 8). There are several signalling pathways known to be involved upon CRTH2 activation and control the varying effects of CRTH2 agonists. Indomethacin induces Ca^2+^ mobilization via CRTH2 in Th2 cells, which is inhibited by pertussin toxin indicating a G_α/i_ dependent mechanism [45]. Indomethacin induced eosinophil morphological change and upregulation of CD11b via CRTH2 can be attenuated with inhibitors of MAP Kinase and phosphatidylinositol 3-kinase (PI3K), suggesting an important role for these signalling pathways in CRTH2 mediated effects [46]. PGD2 induces IL-4, IL-5 and IL-13 production and cell migration in Th2 cells via CRTH2, which is also attenuated by inhibiting the PI3K pathway [47]. The only other study to explore the effect of CRTH2 agonists on NF-κB showed that an IKB inhibitor had no effect PGD2 induced cytokine production, however the NF-κB inhibitor rocaglamide reduced PGD2 induced cytokine production by 30% [47]. The authors of this study concluded that since PGD2 did not lead to an increase phospho-IκB, a marker of NF-κB activation, that this effect was likely to be non-pathway specific. Together with their data showing no direct effect on NF-κB signalling upon CRTH2 activation with PGD2, our data also supports no direct effect on NF-κB signalling with CRTH2 activation or inhibition (Figure 8).

The CRTH2 agonist 15dPGJ2 is an endogenous anti-inflammatory prostaglandin, formed from a non-enzymatic dehydration of PGD2 [48]. It can accumulate intracellularly [49], and can be measured endogenously in picomolar concentrations in body fluids [50]. Although it is a high affinity ligand to the nuclear PPAR-γ receptor, 15dPGJ2 inhibits NF-κB independently of this receptor in amniocytes and myocytes [14]. We have previously shown that 15dPGJ2 is able to inhibit multiple steps in the IKK/NF-κB pathway. It inhibits IL-1β induced phosphorylation of IKKα and IKKβ, prevents IKBα degradation and thus prevents the release of the NF-κB subunits for nuclear translocation [14]. Direct modification of cysteine 179 of IKKβ by covalent interaction by 15dPGJ2 has been demonstrated in HeLa cells [51].

15dPGJ2 contains an electrophilic cyclopenteneone ring, a property which permits it to ligate nuclear receptors and modify intracellular signalling molecules. The highly reactive ring of 15dPGJ2 can form a covalent interaction with several components of the NF-κB signalling pathway (IkB kinase complex B, p50, and p65 subunits) via the Michael reaction, resulting in impaired nuclear entry, as described above, and impaired DNA binding activity [52]. It is highly likely that the cyclopentenone ring is essential for the inhibition of NF-κB seen in PBMCs in this study, since we have previously shown the ability of PGA_1_, but not 9,10-dihydro-15dPGJ2 (in which the ring is disrupted) to mimic the effect of 15dPGJ2 in amniocytes and myocytes.

Despite the above mechanisms having been described, it is not yet clear how 15dPGJ2 enters the cell to reach its targets, or if there is an alternative receptor mediated mechanism. Although Prostaglandin J2 has been shown to enter the cell by active transport [53], it is not yet clear if this is the mechanism of entry of 15dPGJ2 into amniocytes, myocytes and PBMCs. In this study we have excluded a CRTH2 dependent receptor mediated entry into the cell as a requirement for NF-κB inhibition, and CRTH2 dependent downstream signalling effect on the inhibition of NF-κB. Further studies should be carried out to investigate other potential mechanisms of 15dPGJ2 mediated NF-κB inhibition, including mechanism of entry into the cell, and to explore its effect on the production of NF-κB regulated interleukins in amniocytes and myocytes.

### Conclusions

We have demonstrated that despite being able to detect low levels of mRNA in amniocytes and myocytes, neither cell type express CRTH2 at a detectable protein level. We conclude that the mechanism of 15dPGJ2 mediated inhibition of NF-κB does not involve CRTH2. Small molecule CRTH2 agonists are therefore unlikely to be of value in the inhibition of inflammation associated preterm birth.

## References

[1] Beck S, Wojdyla D, Say L, Betran AP, Merialdi M, et al (2010) The worldwide incidence of preterm birth: a systematic review of maternal mortality and morbidity. Bull World Health Organ 88: 31–38. 20428351 10.2471/BLT.08.062554 PMC2802437

[2] Romero R, Mazor M, Munoz H, Gomez R, Galasso M, et al (1994) The preterm labor syndrome. Ann N Y Acad Sci 734: 414–429. 7978942 10.1111/j.1749-6632.1994.tb21771.x

[3] Goldenberg RL, Hauth JC, Andrews WW (2000) Intrauterine infection and preterm delivery. N Engl J Med 342: 1500–1507. 10816189 10.1056/NEJM200005183422007

[4] Sykes L, MacIntyre D, Teoh T, Bennett P (2011) Targeting immune activation in the prevention of preterm labour. European Obstetrics and Gynaecology 6: 100–106.

[5] Lappas M, Rice GE (2009) Transcriptional regulation of the processes of human labour and delivery. Placenta 30 Suppl A: S90–95. 10.1016/j.placenta.2008.10.005 19010537

[6] McCracken SA, Hadfield K, Rahimi Z, Gallery ED, Morris JM (2007) NF-kappaB-regulated suppression of T-bet in T cells represses Th1 immune responses in pregnancy. Eur J Immunol 37: 1386–1396. 17407192 10.1002/eji.200636322

[7] Lindstrom TM, Bennett PR (2005) The role of nuclear factor kappa B in human labour. Reproduction 130: 569–581. 16264088 10.1530/rep.1.00197

[8] Condon JC, Hardy DB, Kovaric K, Mendelson CR (2006) Up-regulation of the progesterone receptor (PR)-C isoform in laboring myometrium by activation of nuclear factor-kappaB may contribute to the onset of labor through inhibition of PR function. Mol Endocrinol 20: 764–775. 16339279 10.1210/me.2005-0242

[9] Chapman NR, Europe-Finner GN, Robson SC (2004) Expression and deoxyribonucleic acid-binding activity of the nuclear factor kappaB family in the human myometrium during pregnancy and labor. J Clin Endocrinol Metab 89: 5683–5693. 15531529 10.1210/jc.2004-0873

[10] Allport VC, Pieber D, Slater DM, Newton R, White JO, et al (2001) Human labour is associated with nuclear factor-kappaB activity which mediates cyclo-oxygenase-2 expression and is involved with the ‘functional progesterone withdrawal’. Mol Hum Reprod 7: 581–586. 11385114 10.1093/molehr/7.6.581

[11] Lim S, Macintyre DA, Lee YS, Khanjani S, Terzidou V, et al (2012) Nuclear factor kappa B activation occurs in the amnion prior to labour onset and modulates the expression of numerous labour associated genes. PLoS One 7: e34707. 22485186 10.1371/journal.pone.0034707 PMC3317641

[12] Lappas M, Rice GE (2007) The role and regulation of the nuclear factor kappa B signalling pathway in human labour. Placenta 28: 543–556. 16843526 10.1016/j.placenta.2006.05.011

[13] Barnes PJ, Karin M (1997) Nuclear factor-kappaB: a pivotal transcription factor in chronic inflammatory diseases. N Engl J Med 336: 1066–1071. 9091804 10.1056/NEJM199704103361506

[14] Lindstrom TM, Bennett PR (2005) 15-Deoxy-{delta}12,14-prostaglandin j2 inhibits interleukin-1{beta}-induced nuclear factor-{kappa}b in human amnion and myometrial cells: mechanisms and implications. J Clin Endocrinol Metab 90: 3534–3543. 15755849 10.1210/jc.2005-0055

[15] Sykes L, MacIntyre DA, Yap X, Ponnampalam S, Teoh TG, et al (2012) Changes in the Th1:Th2 cytokine bias in pregnancy and the effects of the anti-inflammatory cyclopentenone prostaglandin 15-deoxy-Δ 12,14- Prostaglandin J2. Mediators of Inflammation 2012: 1–12. 10.1155/2012/416739 PMC3368617 22690041

[16] Pirianov G, Waddington SN, Lindstrom TM, Terzidou V, Mehmet H, et al (2009) The cyclopentenone 15-deoxy-delta 12,14-prostaglandin J(2) delays lipopolysaccharide-induced preterm delivery and reduces mortality in the newborn mouse. Endocrinology 150: 699–706. 18845626 10.1210/en.2008-1178

[17] Sawyer N, Cauchon E, Chateauneuf A, Cruz RP, Nicholson DW, et al (2002) Molecular pharmacology of the human prostaglandin D2 receptor, CRTH2. Br J Pharmacol 137: 1163–1172. 12466225 10.1038/sj.bjp.0704973 PMC1573602

[18] Nagata K (2003) Crth2. J Biol Regul Homeost Agents 17: 334–337. 15065763

[19] Nagata K, Tanaka K, Ogawa K, Kemmotsu K, Imai T, et al (1999) Selective expression of a novel surface molecule by human Th2 cells in vivo. J Immunol 162: 1278–1286. 9973380

[20] Monneret G, Gravel S, Diamond M, Rokach J, Powell WS (2001) Prostaglandin D2 is a potent chemoattractant for human eosinophils that acts via a novel DP receptor. Blood 98: 1942–1948. 11535533 10.1182/blood.v98.6.1942

[21] Marchese A, Sawzdargo M, Nguyen T, Cheng R, Heng HH, et al (1999) Discovery of three novel orphan G-protein-coupled receptors. Genomics 56: 12–21. 10036181 10.1006/geno.1998.5655

[22] De Fanis U, Mori F, Kurnat RJ, Lee WK, Bova M, et al (2007) GATA3 up-regulation associated with surface expression of CD294/CRTH2: a unique feature of human Th cells. Blood 109: 4343–4350. 17234745 10.1182/blood-2006-05-025940 PMC1885489

[23] Nagata K, Hirai H, Tanaka K, Ogawa K, Aso T, et al (1999) CRTH2, an orphan receptor of T-helper-2-cells, is expressed on basophils and eosinophils and responds to mast cell-derived factor(s). FEBS Lett 459: 195–199. 10518017 10.1016/s0014-5793(99)01251-x

[24] Nagata K, Hirai H (2003) The second PGD(2) receptor CRTH2: structure, properties, and functions in leukocytes. Prostaglandins Leukot Essent Fatty Acids 69: 169–177. 12895600 10.1016/s0952-3278(03)00078-4

[25] Gallant MA, Samadfam R, Hackett JA, Antoniou J, Parent JL, et al (2005) Production of prostaglandin D(2) by human osteoblasts and modulation of osteoprotegerin, RANKL, and cellular migration by DP and CRTH2 receptors. J Bone Miner Res 20: 672–681. 15765187 10.1359/JBMR.041211

[26] Zayed N, Afif H, Chabane N, Mfuna-Endam L, Benderdour M, et al (2008) Inhibition of interleukin-1beta-induced matrix metalloproteinases 1 and 13 production in human osteoarthritic chondrocytes by prostaglandin D2. Arthritis Rheum 58: 3530–3540. 18975308 10.1002/art.23958 PMC2657141

[27] Chiba T, Kanda A, Ueki S, Ito W, Kamada Y, et al (2006) Prostaglandin D2 induces IL-8 and GM-CSF by bronchial epithelial cells in a CRTH2-independent pathway. Int Arch Allergy Immunol 141: 300–307. 16940740 10.1159/000095436

[28] Colombe L, Michelet JF, Bernard BA (2008) Prostanoid receptors in anagen human hair follicles. Exp Dermatol 17: 63–72. 18005048 10.1111/j.1600-0625.2007.00639.x

[29] Gervais FG, Morello JP, Beaulieu C, Sawyer N, Denis D, et al (2005) Identification of a potent and selective synthetic agonist at the CRTH2 receptor. Mol Pharmacol 67: 1834–1839. 15755909 10.1124/mol.104.009068

[30] Terzidou V, Lee Y, Lindstrom T, Johnson M, Thornton S, et al (2006) Regulation of the human oxytocin receptor by nuclear factor-kappaB and CCAAT/enhancer-binding protein-beta. J Clin Endocrinol Metab 91: 2317–2326. 16569740 10.1210/jc.2005-2649

[31] Venet F, Lepape A, Debard AL, Bienvenu J, Bohe J, et al (2004) The Th2 response as monitored by CRTH2 or CCR3 expression is severely decreased during septic shock. Clin Immunol 113: 278–284. 15507393 10.1016/j.clim.2004.07.005

[32] El-Shazly AE, Moonen V, Mawet M, Begon D, Henket M, et al (2011) IFN-gamma and TNF-alpha potentiate prostaglandin D2-induced human eosinophil chemotaxis through up-regulation of CRTH2 surface receptor. Int Immunopharmacol 11: 1864–1870. 21835268 10.1016/j.intimp.2011.07.017

[33] Wen SW, Smith G, Yang Q, Walker M (2004) Epidemiology of preterm birth and neonatal outcome. Semin Fetal Neonatal Med 9: 429–435. 15691780 10.1016/j.siny.2004.04.002

[34] Goldenberg RL, Andrews WW, Hauth JC (2002) Choriodecidual infection and preterm birth. Nutr Rev 60: S19–25. 12035853 10.1301/00296640260130696

[35] Gotsch F, Romero R, Kusanovic JP, Mazaki-Tovi S, Pineles BL, et al (2007) The fetal inflammatory response syndrome. Clin Obstet Gynecol 50: 652–683. 17762416 10.1097/GRF.0b013e31811ebef6

[36] Monneret G, Li H, Vasilescu J, Rokach J, Powell WS (2002) 15-Deoxy-delta 12,14-prostaglandins D2 and J2 are potent activators of human eosinophils. J Immunol 168: 3563–3569. 11907120 10.4049/jimmunol.168.7.3563

[37] Helliwell RJ, Keelan JA, Marvin KW, Adams L, Chang MC, et al (2006) Gestational age-dependent up-regulation of prostaglandin D synthase (PGDS) and production of PGDS-derived antiinflammatory prostaglandins in human placenta. J Clin Endocrinol Metab 91: 597–606. 16291703 10.1210/jc.2005-1982

[38] Tsuda H, Michimata T, Sakai M, Nagata K, Nakamura M, et al (2001) A novel surface molecule of Th2- and Tc2-type cells, CRTH2 expression on human peripheral and decidual CD4+ and CD8+ T cells during the early stage of pregnancy. Clin Exp Immunol 123: 105–111. 11168006 10.1046/j.1365-2249.2001.01422.x PMC1905966

[39] Michimata T, Tsuda H, Sakai M, Fujimura M, Nagata K, et al (2002) Accumulation of CRTH2-positive T-helper 2 and T-cytotoxic 2 cells at implantation sites of human decidua in a prostaglandin D(2)-mediated manner. Mol Hum Reprod 8: 181–187. 11818521 10.1093/molehr/8.2.181

[40] Michimata T, Sakai M, Miyazaki S, Ogasawara MS, Suzumori K, et al (2003) Decrease of T-helper 2 and T-cytotoxic 2 cells at implantation sites occurs in unexplained recurrent spontaneous abortion with normal chromosomal content. Hum Reprod 18: 1523–1528. 12832382 10.1093/humrep/deg280

[41] Gosset P, Bureau F, Angeli V, Pichavant M, Faveeuw C, et al (2003) Prostaglandin D2 affects the maturation of human monocyte-derived dendritic cells: consequence on the polarization of naive Th cells. J Immunol 170: 4943–4952. 12734337 10.4049/jimmunol.170.10.4943

[42] Durand M, Gallant MA, de Brum-Fernandes AJ (2008) Prostaglandin D2 receptors control osteoclastogenesis and the activity of human osteoclasts. J Bone Miner Res 23: 1097–1105. 18302497 10.1359/jbmr.080228

[43] Cosmi L, Annunziato F, Galli MIG, Maggi RME, Nagata K, et al (2000) CRTH2 is the most reliable marker for the detection of circulating human type 2 Th and type 2 T cytotoxic cells in health and disease. Eur J Immunol 30: 2972–2979. 11069080 10.1002/1521-4141(200010)30:10<2972::AID-IMMU2972>3.0.CO;2-#

[44] Iwasaki M, Nagata K, Takano S, Takahashi K, Ishii N, et al (2002) Association of a new-type prostaglandin D2 receptor CRTH2 with circulating T helper 2 cells in patients with atopic dermatitis. J Invest Dermatol 119: 609–616. 12230502 10.1046/j.1523-1747.2002.01862.x

[45] Hirai H, Tanaka K, Takano S, Ichimasa M, Nakamura M, et al (2002) Cutting edge: agonistic effect of indomethacin on a prostaglandin D2 receptor, CRTH2. J Immunol 168: 981–985. 11801628 10.4049/jimmunol.168.3.981

[46] Stubbs VE, Schratl P, Hartnell A, Williams TJ, Peskar BA, et al (2002) Indomethacin causes prostaglandin D(2)-like and eotaxin-like selective responses in eosinophils and basophils. J Biol Chem 277: 26012–26020. 11980903 10.1074/jbc.M201803200

[47] Xue L, Gyles SL, Barrow A, Pettipher R (2007) Inhibition of PI3K and calcineurin suppresses chemoattractant receptor-homologous molecule expressed on Th2 cells (CRTH2)-dependent responses of Th2 lymphocytes to prostaglandin D(2). Biochem Pharmacol 73: 843–853. 17196174 10.1016/j.bcp.2006.11.021

[48] Fitzpatrick FA, Wynalda MA (1983) Albumin-catalyzed metabolism of prostaglandin D2. Identification of products formed in vitro. J Biol Chem 258: 11713–11718. 6578214

[49] Shibata T, Kondo M, Osawa T, Shibata N, Kobayashi M, et al (2002) 15-deoxy-delta 12,14-prostaglandin J2. A prostaglandin D2 metabolite generated during inflammatory processes. J Biol Chem 277: 10459–10466. 11786541 10.1074/jbc.M110314200

[50] Bell-Parikh LC, Ide T, Lawson JA, McNamara P, Reilly M, et al (2003) Biosynthesis of 15-deoxy-delta12,14-PGJ2 and the ligation of PPARgamma. J Clin Invest 112: 945–955. 12975479 10.1172/JCI18012 PMC193665

[51] Rossi A, Kapahi P, Natoli G, Takahashi T, Chen Y, et al (2000) Anti-inflammatory cyclopentenone prostaglandins are direct inhibitors of IkappaB kinase. Nature 403: 103–108. 10638762 10.1038/47520

[52] Cernuda-Morollon E, Pineda-Molina E, Canada FJ, Perez-Sala D (2001) 15-Deoxy-Delta 12,14-prostaglandin J2 inhibition of NF-kappaB-DNA binding through covalent modification of the p50 subunit. J Biol Chem 276: 35530–35536. 11466314 10.1074/jbc.M104518200

[53] Narumiya S, Ohno K, Fukushima M, Fujiwara M (1987) Site and mechanism of growth inhibition by prostaglandins. III. Distribution and binding of prostaglandin A2 and delta 12-prostaglandin J2 in nuclei. J Pharmacol Exp Ther 242: 306–311. 3302206

